# Female Genital Mutilation and Obstetric Outcomes: Flawed Systematic Review and Meta-Analysis Does Not Accurately Reflect the Available Evidence

**DOI:** 10.1155/2014/205230

**Published:** 2014-03-20

**Authors:** O. Meirik, E. Banks, T. Farley, O. Akande, H. Bathija, M. Ali

**Affiliations:** ^1^Instituto Chileno de Medicina Reproductiva (ICMER), 8320165 Santiago, Chile; ^2^National Centre for Epidemiology and Population Health, Australian National University, 0200 Canberra, Australia; ^3^Sigma3 Services SARL, Scientific & Statistical Solutions, 1260 Nyon, Switzerland; ^4^College of Medicine, University College Hospital, Ibadan 1001, Nigeria; ^5^Geneva Foundation for Medical Education and Research, 1290 Geneva, Switzerland; ^6^Regional Office for the Eastern Mediterranean, World Health Organization, Cairo 11371, Egypt

We commend Berg and Underland for taking on the momentous task of systematically reviewing and summarizing available data on the association between female genital mutilation (FGM) and obstetric outcomes [1, 2]. FGM is an important health and human rights issue and reliable evidence on its effects on health is critical for advocacy to encourage its abandonment.

Despite the obvious hard work and adherence to a prespecified protocol, there are two major problems with this systematic review that undermine the validity of the conclusions reached.

The first problem is that the review did not distinguish between studies of fundamentally differing designs and combined their results to reach summary estimates. The studies included in the Berg and Underland review were from a wide variety of countries and all were observational [1]. Three of the studies included in the review ascertained FGM status prior to the occurrence of delivery and followed the study participants for their outcomes at delivery [3–5]. The rest of the studies ascertained FGM status and obstetric outcomes at the same time, often retrospectively. However, all studies were classified as cross-sectional and their results were combined. The authors claim that there is a lack of cohort study data (i.e., prospective data) on FGM, hence difficulties coming to conclusions about causality, despite the existence of the three studies with prospectively ascertained FGM and follow-up for outcomes, including the UNDP/UNFPA/World Bank/World Health Organization Special Programme on Research Development and Research Training in Human Reproduction (WHO/HRP) study outlined below [3]. The evidence contained in the higher quality studies is effectively obscured by the lower quality data from the more numerous cross-sectional studies.

The second is that crude results were used to calculate summary estimates of the relationship of FGM to obstetric outcomes, even when appropriately adjusted results were available. The obstetric outcomes examined in the review include prolonged labor, obstetric tears/lacerations, Caesarean section, episiotomy, instrumental delivery, obstetric/postpartum haemorrhage, and difficult labor/dystocia. The operational definitions of several of these endpoints differ across countries and between hospitals within countries, as do the frequencies of the endpoints related to interventions during delivery, since these depend on prevailing, often hospital-specific obstetric practices. The prevalence of the different types of FGM varies widely within and between countries. The occurrence of these exposures and outcomes are also likely to vary according to participant factors such as age and parity. Therefore, a range of confounding factors are likely to be important in assessing the relationship of FGM to obstetric outcome in the data used for this systemic review and meta-analysis. However, the authors used RevMan v5.2.4 [6], which is a program largely designed to deal with randomized controlled trials, to calculate summary risk ratios (RR) from crude numbers and hence did not account for potential confounding factors and did not use adjusted estimates of risk, even when these were published.

To illustrate the pitfalls of using this inappropriate methodology for meta-analysis of observational data, we used data from the study conducted by WHO/HRP on obstetric outcomes in women exposed to FGM [3], which is among the studies included in the review by Berg and Underland [1, 2].

We compared the adjusted relative risk (RR) estimates from the WHO/HRP study in the original study publication in the Lancet with the RR estimates for the WHO/HRP study calculated by Berg and Underland from crude data, pooling all types of FGMs into one “exposed” group (see Table 1). The WHO/HRP study was conducted in 28 hospitals in 6 countries in Africa and ascertained the type of FGM by clinical examination in individual women prior to delivery, along with data on a range of potential confounding factors. The means of identifying and measuring potential confounding factors is explained in the original publication of the WHO/HRP study [3]. The four crude estimates calculated by Berg and Underland differ substantively from the original adjusted WHO/HRP results, and for two of them, namely, perineal tears and Caesarean section, the crude results suggest, erroneously, that FGM protects against the examined obstetric outcome, directly contrary to the adjusted relative risk estimates in the original Lancet publication [3] (see Table 1). The use of crude numbers to estimate these relative risks from multicentre observational studies is incorrect and produces misleading results.

Berg and Underland refer to a dose-response relationship as an important factor in determining a causal relationship but fail to consider it when deliberating on the issue of causality between FGM and poor obstetric outcome. In the WHO/HRP study, there was a dose-response relationship between the severity of the FGM (from FGM type l through type lll) and the magnitude of the relative risk estimate for virtually all examined obstetric outcomes (Caesarean section, postpartum haemorrhage, extended maternal hospital stay, resuscitation of infant, inpatient perinatal death, fresh stillbirth, episiotomy, and vaginal tear), except for risk of an infant with low birth weight (LBW), macerated stillbirth, and Apgar score < 4. This pattern of dose-response was similar among parous and nulliparous women.

Based on the findings of their meta-analysis and review, Berg and Underland conclude that “the quality of the evidence for all outcomes as being too low to warrant conclusions about a causal relationship between FGM/C and obstetric complications” and that “inconsistencies in results and estimate imprecision” contribute to this conclusion [1]. By using erroneous statistical methods for meta-analysis and inappropriately combining the results of disparate study designs, it is not surprising that the authors arrived at this bland conclusion, which does not do justice to the available evidence. The evidence indicates that the risk of many adverse obstetric outcomes is increased in women who have had FGM, compared to those who have not had it, and that this relationship is likely to be causal.

## Figures and Tables

**Table 1:** Relative risk (RR) estimates (95% confidence limits) for health outcomes in a data set on obstetric outcome of delivery by FGM status and method of analysis. Adjusted RRs from WHO/HRP 2006 and unadjusted RRs from Berg and Underland 2013.

Health outcomes and comparison groups	WHO/HRP original published results (adjusted^1^ RR (95% CI) by type of FGM and parity) [3]	WHO/HRP results presented by Berg and Underland (unadjusted RR (95% CI), all types of FGM) [1, 2]
Perineal tear	Primiparous women^2^	All women 0.90 (0.82–1.00)
FGM I versus no FGM	1.31 (1.03–1.66)	
FGM II versus no FGM	1.92 (1.50–2.47)	
FGM III versus no FGM	3.19 (1.91–4.74)	
	Multiparous women^2^	
FGM I versus no FGM	1.37 (1.07–1.75)	
FGM II versus no FGM	2.17 (1.69–2.82)	
FGM III versus no FGM	1.93 (1.07–3.38)	

Episiotomy	Primiparous women^3^	All women 1.57 (1.51–1.63)
FGM I versus no FGM	1.31 (1.20–1.44)	
FGM II versus no FGM	1.47 (1.34–1.60)	
FGM III versus no FGM	1.84 (1.70–1.97)	
	Multiparous women^3^	
FGM I versus no FGM	1.75 (1.47–2.09)	
FGM II versus no FGM	2.02 (1.69–2.42)	
FGM III versus no FGM	2.16 (1.91–2.44)	

Caesarean section	All women	All women 0.83 (0.75–0.91)
FGM I versus no FGM	1.03 (0.88–1.21)	
FGM II versus no FGM	1.29 (1.09–1.52)	
FGM III versus no FGM	1.31 (1.01–1.70)	

Postpartum haemorrhage	All women	All women 1.23 (1.11–1.36)
FGM I versus no FGM	1.03 (0.87–1.21)	
FGM II versus no FGM	1.21 (1.01–1.43)	
FGM III versus no FGM	1.69 (1.34–2.12)	

^1^Adjusted for study centre, maternal age, parity, maternal height, maternal education, socioeconomic status, residence (urban or rural), time taken to reach hospital, and number of antenatal care visits. ^2^Women without episiotomy. ^3^Women with or without a perineal tear.
